# Effect of Perioperative Tranexamic Acid on Allogeneic Blood Transfusions for Total Knee Arthroplasty Patients at a Community Hospital

**DOI:** 10.7759/cureus.13951

**Published:** 2021-03-17

**Authors:** Chukwuweike Gwam, Kylie Kroes, Kevin Wang, Arianne Wilson, Daniel P Bullock, Bartlomiej W Szczech

**Affiliations:** 1 Department of Orthopedic Surgery, Wake Forest Baptist Health, Winston Salem, USA; 2 Department of Biology, Colgate University, Hamilton, USA; 3 Department of Orthopedic Surgery, Wake Forest Baptist Medical Center, Winston Salem, USA; 4 Department of Family Medicine, MedStar Health/Georgetown-Washington Hospital, Washington DC, USA; 5 Orthopedics, Lake Placid Sports Medicine, Lake Placid, USA

**Keywords:** tranexamic acid, community hospital, primary knee replacement, txa

## Abstract

Introduction

Tranexamic acid (TXA) has been shown to be a cost-effective method for reducing blood loss and postoperative transfusions in patients undergoing total knee arthroplasty (TKA) at tertiary care centers. However, the efficacy of TXA has not been studied in community hospitals, and the potential cost savings may be especially beneficial for these institutions. The purpose of this study was to assess the effectiveness of TXA in reducing postoperative transfusions and blood loss following TKA at a community hospital.

Methods

Institutional approval was obtained for the retrospective review of a consecutive series of patients that underwent a total knee arthroplasty procedure between January 1, 2019, and December 31, 2019. Patients undergoing bilateral TKA were excluded from the analysis, yielding a total of 190 TKA procedures of which 131 patients received TXA. Fisher's exact test was conducted to compare rates of transfusion between the groups. A difference in difference analysis was conducted to assess TXA’s effect on patient hemoglobin levels (Hgb) on postoperative Days 1 and 2. All analyses were conducted using R studio (Vienna, Austria). A p-value of 0.05 was set as the threshold for statistical significance.

Results

There was no difference in group characteristics in terms of age (70 years vs 68 years, p=0.17; no-TXA vs TXA, respectively). Fisher's exact test revealed no difference in the rates of allogeneic transfusion between TKA patients who did not receive a TXA and TKA patients who received a TXA (3.4% vs 0.8%; p=0.228). However, our difference in differences analysis revealed that TXA patients had a mean reduction in hemoglobin (Hgb)-related blood loss of 0.876 Hgb/dl (95% CI: 0.56 to 1.19; p<0.001) between the preoperative period and postoperative Day 1. Similarly, our difference in differences analysis revealed a mean reduction in Hgb-related blood loss of 0.972 Hgb/dl (95% CI: 0.593 to 1.349; p<0.001) between the preoperative period and postoperative Day 2.

Conclusion

The present study shows TXA to be effective for reducing blood loss and transfusions following TKAs performed at a small community hospital. Given the cost-savings previously reported with TXA use, as well as the medical benefits reported in this study, TXA may have a niche in small community hospitals where cost savings from reduced transfusions and shorter hospital stays are important. Further studies should assess the exact amount of financial savings from TXA utilization in small community hospitals.

## Introduction

Over 750,000 total knee arthroplasties (TKA) are performed in the United States each year, and the number is projected to increase by 143% by 2050 [1-3]. Although TKA is commonly regarded as one of the most successful surgical procedures, it is not without risk. Recent studies have reported complications following TKA, ranging from 1.7% to 5.6%, with blood loss and subsequent postoperative transfusion rates being two of the most important complications to consider perioperatively [4-5]. To combat blood loss and postoperative transfusion requirement, there has been a trend toward using both topical and intravenous (IV) tranexamic acid (TXA) over the past decade [6-8].

TXA is a commonly used antifibrinolytic agent that has been shown to reduce blood loss and postoperative transfusions in various orthopedic procedures. Although prior literature has proven the efficacy of TXA in the tertiary care setting, there is a paucity of literature concerning the use of TXA at community hospitals, where resources may not be as available as in larger hospital settings. A variety of factors may prevent community hospitals from achieving the quality of care provided by larger hospital systems, including not having the newest medical innovations, shortages of physicians and other healthcare professionals, as well as a lack of comprehensive subspecialties that can limit coordinated care efforts.

Since TXA is a low-cost method for reducing transfusions, it may be optimal for improving the quality of care in the community hospital setting. The purpose of this study was to determine the relationship between TXA and allogeneic blood transfusion rates following TKA in a small community hospital.

## Materials and methods

Patient population

This retrospective analysis consisted of collecting and analyzing data from all patients who underwent a TKA procedure performed by four orthopedic surgeons at a community hospital between January 1, 2019, and December 31, 2019. Inclusion criteria included a preoperative diagnosis of end-stage tri-compartmental osteoarthritis. Exclusion criteria included patients who had a primary diagnosis of inflammatory arthropathy, avascular necrosis, and patients undergoing simultaneous bilateral TKA. All TKA surgeries in the analysis underwent the same standard midline parapatellar approach. Additionally, all patients underwent the same preoperative pathway and patients underwent either spinal or general anesthesia based on the anesthesiologists' preoperative assessment.

TXA use

Intravenous TXA use was based on surgeon preference. For example, one surgeon always used TXA when performing a TKA, whereas another surgeon in the practice never did. The other surgeons' use of TXA varied based on the patient and the surgeon's judgment. All ages and both sexes were included in this study, leading to significant variation when it came to the population of 222 individuals analyzed. The mean patient age was 68 years (range, 49 to 91 years).

Statistical analysis

For statistical analysis, 95% confidence intervals were calculated, and significance was set at a level of 0.001 when calculating P-values. Continuous variables were analyzed with a paired t-test. Categorical variables were analyzed via the chi-square test or Fisher's exact test where appropriate. A post-hoc power analysis was performed revealing that 87 patients were needed for medium effect size and 784 for small effect size. All analysis was conducted using R studio software (Vienna, Austria).

## Results

**Figure 1:**
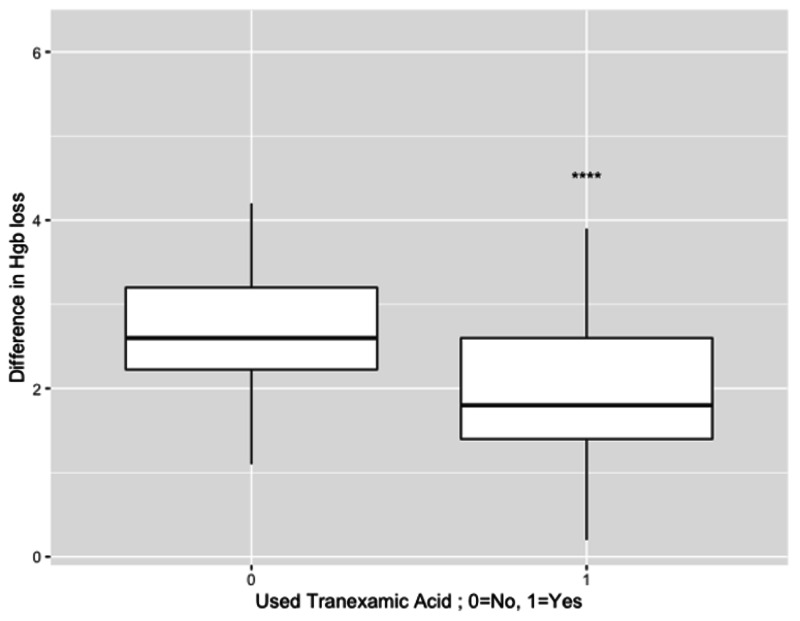
Mean loss of Hgb without TXA=2.806, mean loss with TXA=1.93 95%CI for mean difference=0.564 to 1.185; p<0.001 for preoperative vs postoperative Day 1 ****=p<0.001 Hgb: hemaglobin; TXA: tranexamic acid

There was no difference in group characteristics in terms of mean age (70 years (standard deviation, SD= 9) vs 68 years (SD=9), p=0.17; no-TXA vs TXA, respectively). Fisher's exact test revealed no difference in the rates of allogeneic transfusion between TKA patients who did not receive TXA and TKA patients who received TXA (3.4% vs 0.8%; p=0.228). However, our difference in differences analysis revealed a mean reduction in Hgb-related blood loss of 0.876 Hgb/dl (95% CI: 0.56 to 1.19; p<0.001; Figure 1) between the preoperative period and postoperative Day 1. The mean loss of Hgb between preoperative and postoperative Day 1 without TXA was 2.806 Hgb/dl, whereas the mean loss of Hgb with TXA was 1.93 Hgb/dl (Figure 1).

**Figure 2:**
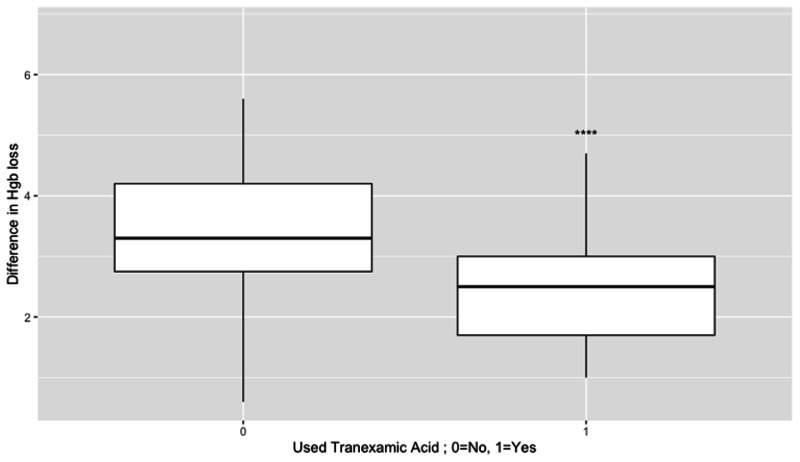
Mean loss of Hgb without TXA=3.438, mean loss with TXA=2.466 95%CI for mean difference= 0.593 to 1.349; p<0.001 for preoperative vs postoperative day 2 ****=p<0.0001 Hgb: hemaglobin; TXA: tranexamic acid

Similarly, our difference in differences analysis revealed a mean reduction in Hgb-related blood loss of 0.972 Hgb/dl (95% CI: 0.593 to 1.349; p<0.001; Figure 2) between the preoperative period and postoperative Day 2. The mean loss of Hgb between preoperative and postoperative Day 2 without TXA was 3.438 Hgb/dl, whereas the mean loss of Hgb with TXA was 2.466 Hgb/dl (Figure 2).

## Discussion

Although TKA is a fairly safe procedure, it is not immune to the potential for blood loss that is present with any operation. To reduce this risk of blood loss, there has been a trend toward increased utilization of TXA for TKAs. The present study found a significant association between TXA and reduced blood loss as well as postoperative transfusion rate in a small community hospital. Furthermore, we demonstrate that the use of TXA reduced the drop in hemoglobin at both postoperative Days 1 and 2. These results carry validity because there were three surgeons that used TXA, either sporadically or regularly, whereas one surgeon did not. Hence, there was variation between patients who did or did not receive treatment, and the use of TXA showed a reduction of Hgb levels and thus blood loss.

Pharmacologically, TXA is a synthetic amino acid that acts as an antifibrinolytic to stabilize clots and reduce active bleeding [9]. Over the last decade, TXA has found a niche within orthopedics, as orthopedic procedures are often fairly invasive and have increased risk for blood loss and subsequent transfusions. In TKAs in particular, total blood loss has been estimated to be 663 mL for primary TKAs [10]. Blood loss following TKA is viewed as a predictable complication, so many different methods have been utilized to decrease this risk of blood loss. These methods include preoperative medical optimization, synovectomy, and pharmacologic agents such as TXA [6,11-12].

In addition to its medical benefits, TXA has been shown to reduce hospital costs associated with orthopedic procedures. Increasing the use of TXA may be of economic benefit at community hospitals as well. Evangelista et al. reported that TXA decreased total hip arthroplasty transfusions from 22.7% to 11.9%, and TKA from 19.4% to 7.0%, leading to an average hospital cost reduction for total hip arthroplasty (THA) and TKA of $3083 and $2582, respectively [13]. Tuttle et al. similarly demonstrated a reduction in transfusion costs of $83.73 per patient after accounting for the upfront cost of TXA [14]. Taken together, there is substantial evidence that TXA may reduce costs in the community hospital setting.

There is a particular focus on identifying areas of cost-saving in medical settings such as small community hospitals where resources and funding may be low [15-16]. The present study assessed TXA use and effectiveness specifically within a small community hospital, which is the type of medical center where cost-savings is likely of high importance. Since our study found that IV TXA is effective at reducing both blood loss and perioperative blood transfusions, it is likely that increased use of TXA in the hospital assessed in our study, as well as in similar medical centers around the nation, will reduce the ultimate costs associated with TKA procedures. This is especially important for high-utilization procedures like TKA, which are commonly performed in community-based and rural medical settings.

Interestingly, a recent review by Ward et al. found that rural areas served by community hospitals had higher rates of TKA as compared to urban areas served by larger hospital systems [17]. The authors hypothesize that this may be due to surgical care, such as arthroplasty, being used as a substitute for conservative care in areas where travel to office visits is a barrier to conservative care. In addition, there may be fewer non-orthopedic caregivers such as primary care physicians and rehabilitation specialties. If this is the case, increasing the use of TXA at community hospitals may improve the quality of perioperative care for a large population of patients.

The results of this study should be interpreted within the context of its limitations. First, our study only assessed TKAs performed by four orthopedic surgeons at one small community hospital, so these findings may not be generalizable to community hospitals overall. However, our study is the first to assess TXA use in a small community hospital, which is an important strength of our study given the economic implications of increased TXA use for community hospitals. Second, we were only able to assess the impact of TXA on blood loss and transfusion rates, and we are unable to comment on the efficacy of other modes of TXA administration in small community hospitals. Finally, we did not perform a direct cost analysis, so we are unable to make direct comments on the cost savings associated with TXA use in smaller hospitals. However, it is reasonable to suspect that the cost savings from TXA use would be at least as beneficial for smaller hospitals compared to larger, more funded medical institutions.

## Conclusions

The present study shows TXA to be effective for reducing blood loss and transfusions following TKA. Given the cost-savings previously reported with TXA use, as well as the medical benefits reported in this study, TXA may have a niche in small community hospitals where cost-savings from reduced transfusions and shorter hospital stays are important. Further studies should assess the exact amount of financial savings from TXA utilization in small community hospitals.
